# Supplementary material for: The cryo-EM structure of the human uromodulin filament core reveals a unique assembly mechanism
Source: eLife. 2020 Aug 20;9:e60265. doi: 10.7554/eLife.60265 (PMC7486124; doi:10.7554/eLife.60265)
Supplement: Supplementary file 1. — (A) ZP module linker and interface amino acid positions with known variants (gnomAD). (B) Cryo-EM data collection and model refinement statistics. (C) Flowchart for cryoSPARC cryo-EM processing. (D) Flowchart for cisTEM cryo-EM processing. [file elife-60265-supp1.pdf]

Supplementary file 1A

Table S1: UMOD ZP module residues from the interfaces I_A, I_B, or the linker region in the filament core and their gnomAD variants

UMOD Domain	Position	Interface/CS	Variant Amino Acid
**ZPN**	Y402	A	-
**ZPN**	Y407	B	-
**ZPN**	I413	B	L
**ZPN**	I414	B	-
**ZPN**	R415	B	C, H
**ZPN**	Y427	A	-
**Linker**	L429	A	M
**Linker**	D430	CS_A	N
**Linker**	M431	CS_A	V
**Linker**	K432	CS_A	-
**Linker**	V433	CS_A	I
**Linker**	S434	CS_A	-
**Linker**	L435	CS_A	-
**Linker**	K436	CS_A	-
**Linker**	T437	CS_A	S
**Linker**	A438	CS_A	S
**Linker**	L439	CS_A	-
**Linker**	Q440	CS_A	E, H
**Linker**	P441	B	-
**Linker**	M442	B	-
**Linker**	V443	B	-
**Linker**	S444	B	T
**Linker**	A445	CS_B	V
**Linker**	L446	CS_B	V
**Linker**	N447	CS_B	-
**Linker**	I448	CS_B	-
**Linker**	R449	CS_B	-
**Linker**	V450	CS_B	-
**Linker**	G451	CS_B	-
**Linker**	G452	CS_B	R
**Linker**	T453	CS_B	-
**Linker**	G454	A	S
**Linker**	M455	A	-
**Linker**	F456	A	-
**ZPC**	L491	A	-
**ZPC**	F499	A	L
**ZPC**	Y520	B	-
**ZPC**	D532	B	Y
**ZPC**	F553	B	-
**ZPC**	R554	B	W, Q
**ZPC**	F555	B	-
**ZPC**	Y559	B	D
**ZPC**	D560	B	E
**ZPC**	L570	A	-
**CCS**	R586	-	-
**CTP**	L599	CTP/ZPC	-
**CTP**	L601	CTP/ZPC	F
**CTP**	I604	CTP/ZPC	-

ZP module linker and interface amino acid positions with known variants (gnomAD).

Amino acid variants for each amino acid present in the linker region or at either I_A or I_B are listed, according to the gnomAD database. CCS = consensus cut site for hepsin.

Supplementary file 1B

	Session 1	Session 2
Microscope and camera	Titan Krios (Thermo Fisher Scientific), Cs=2.7 mm; Gatan K2 Summit	
Magnification	130 000	
Voltage (kV)	300	
Data acquisition software	EPU	
Exposure navigation	Image shift	
Collected movies	4679	4864
Electron exposure	45	
Defocus range (µm)	-1.2 to -3.3	-0.8 to -2
Number of frames	40	40
Electron dose ($e^-/Å^2/s$)	7.5	7.5
Exposure time (s)	6	
Pixel size (Å)	1.084	1.084
**Refinement**	UMOD (EMD: 11388, PDB: 6ZS5)	UMOD (EMD: 11389)
Software used	cryoSPARC 2 v2.15	cisTEM 1.0.0 beta
Symmetry imposed	C1	
Picking method	Topaz	Manual picking
Initial particle images (no.)	1.31 M	485 k
Final particle images (no.)	145 k	330 k
Map resolution (Å)	3.47	4.60
FSC threshold	0.143	
Map resolution range (Å)	3.2-7.2	4.4-5.5
**Model statistics**	UMOD AU (6ZS5)	Extended UMOD (6ZYA)
Model resolution (Å)	3.99	
FSC threshold	0.5	
Map sharpening B factor ($Å^2$)	-99.9	
**Model Composition**		
Total atoms	2036	6114
Protein residues	257	772
Mean B factor protein ($Å^2$)	78.79	234.35
Mean B factor ligand ($Å^2$)	130.15	280.53
**R.m.s. deviations**		
Bond lengths (Å)	0.008	0.015
Bond angles (°)	1.214	1.394
Validation		
MolProbity score	1.52	1.75
Clashscore	4.02	7.78
Rotamer outliers (%)	0.0	0.0
**Ramachandran plot**[1]		
Favored (%)	95.26	95.29
Allowed (%)	4.74	4.71
Disallowed (%)	0	0

Cryo-EM data collection and model refinement statistics.

[1]Ramachandran restrains were used during the refinement.

Supplementary file 1C

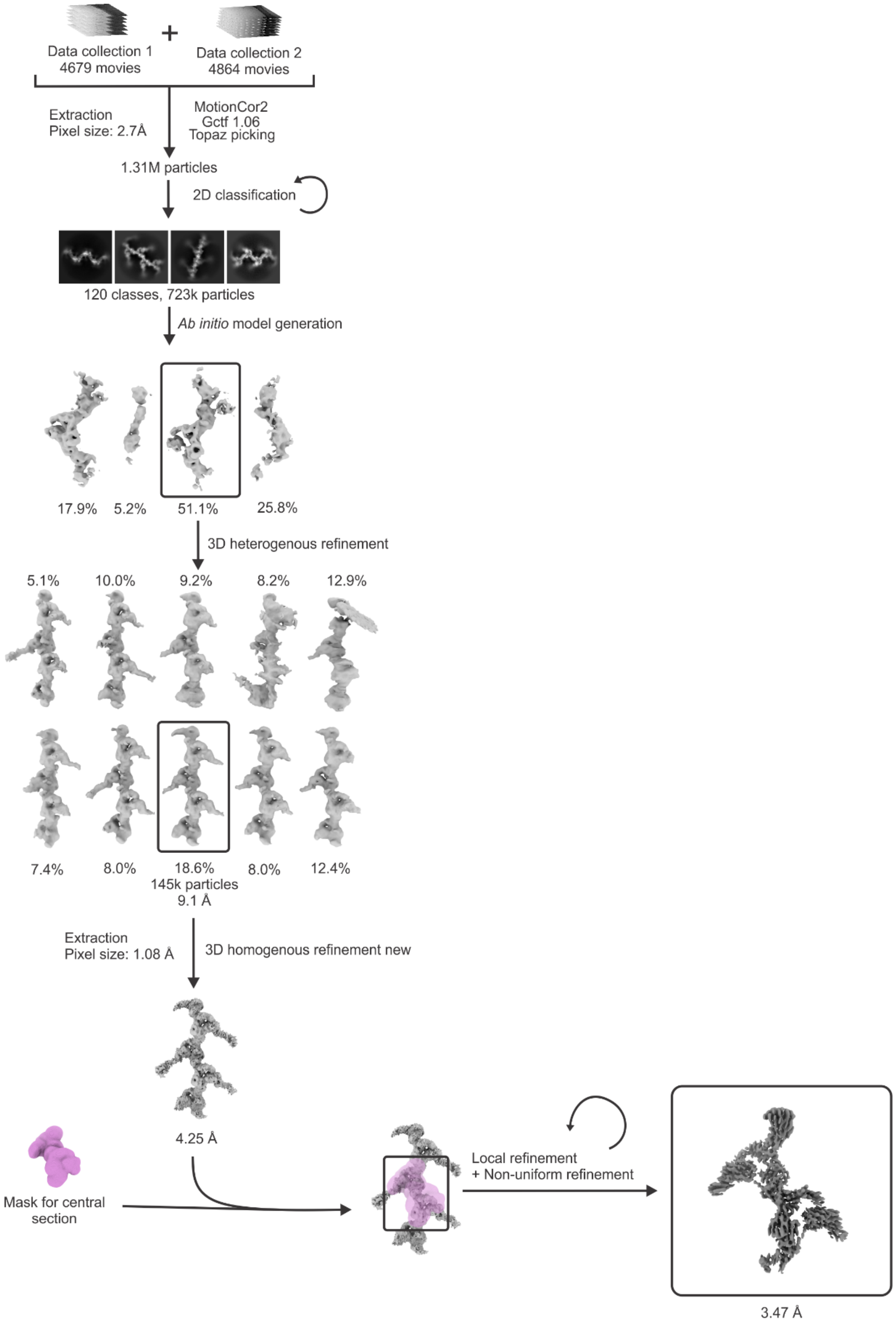

Flowchart for cryoSPARC cryo-EM processing.

Supplementary file 1D

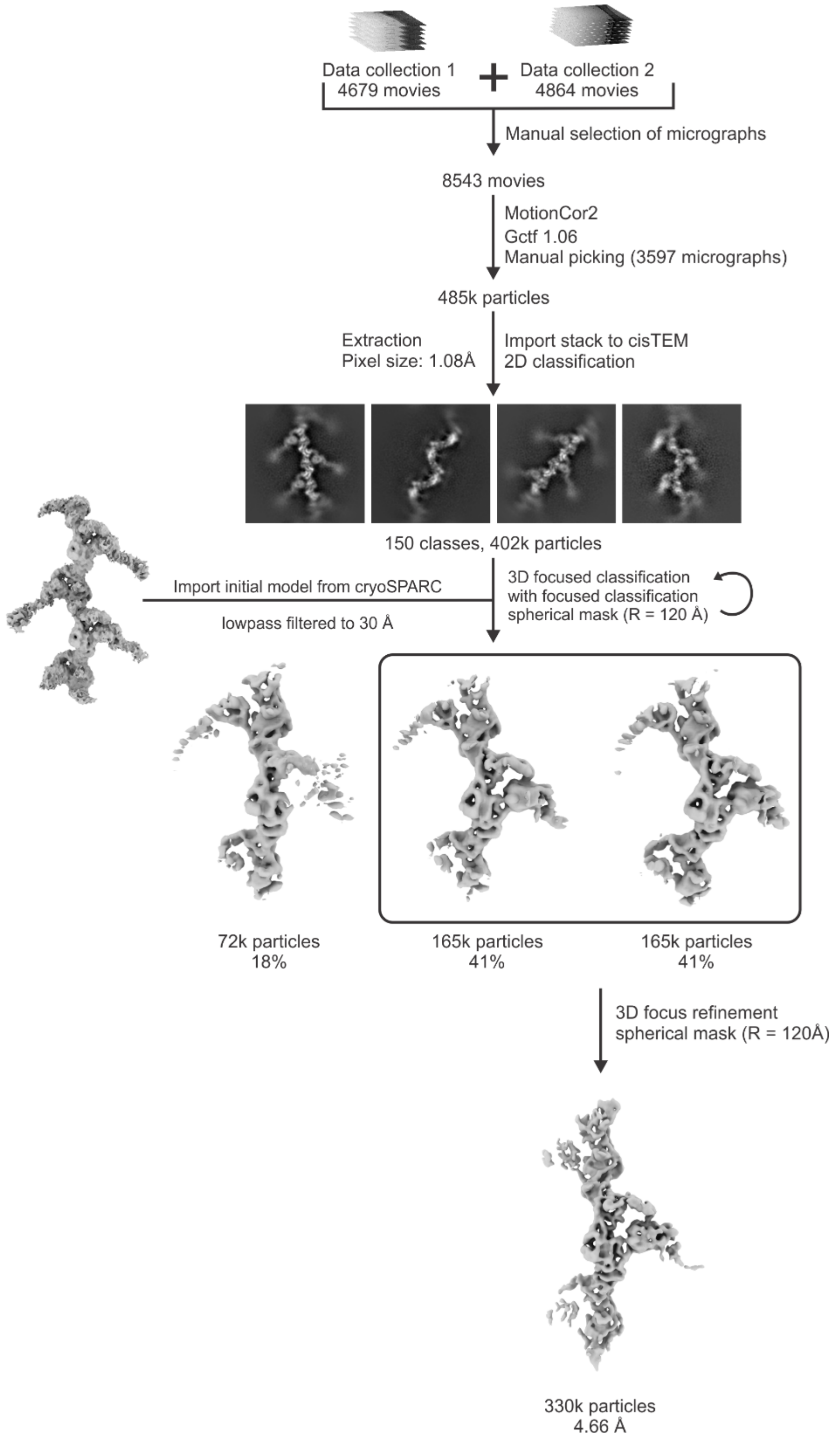

Flowchart for cisTEM cryo-EM processing.
